# BWS-Net: An Optimal Deep Learning Architecture for the Anterior Bladder Wall Segmentation Using Ultrasound Imaging

**DOI:** 10.1109/JBHI.2026.3675965

**Published:** 2026-10

**Authors:** Manali Saini, Yifei Jiang, Tuhinangshu Gangopadhyay, David P. Rosen, Azra Alizad, Mostafa Fatemi

**Affiliations:** Department of Radiology, Mayo Clinic College of Medicine and Science, Rochester, MN 55905 USA; Department of Physiology and Biomedical Engineering, Mayo Clinic College of Medicine and Science, Rochester, MN 55905 USA. He is now with the Department of Biomedical Engineering, University of Michigan, Ann Arbor, MI 48109 USA; Department of Physiology and Biomedical Engineering, Mayo Clinic College of Medicine and Science, Rochester, MN 55905 USA. He is now with the Fraunhofer Institute for Experimental Software Engineering, 67663 Kaiserslautern, Germany; Department of Physiology and Biomedical Engineering, Mayo Clinic College of Medicine and Science, Rochester, MN 55905 USA; Department of Radiology and Department of Physiology and Biomedical Engineering, Mayo Clinic College of Medicine and Science, Rochester, MN 55905 USA; Department of Physiology and Biomedical Engineering, Mayo Clinic College of Medicine and Science, Rochester, MN 55905 USA

**Keywords:** Bladder wall, deep learning, segmentation, ultrasound, artificial intelligence

## Abstract

Urodynamic tests are used to assess bladder function by measuring detrusor pressure, which requires invasive catheterization. Ultrasound bladder vibrometry offers a non-invasive approach to evaluate bladder compliance for diagnostic purposes by estimating detrusor pressure from ultrasound-generated Lamb waves in the bladder wall. Therefore, it is crucial to precisely segment the anterior portion of bladder wall prior to these assessments. Traditional segmentation methods are time-consuming and require manual intervention, while deep learning offers a promising alternative. Existing deep learning approaches are limited and often lack clinical validation. Therefore, we propose a novel deep learning network for precise segmentation of anterior bladder wall. It comprises blueprint separable convolutional layers in an encoder-decoder structure with adaptive attention-based skip connections. Performance evaluation on 8592 distinct images acquired from 64 patients using 5-fold cross-validation demonstrates that it achieves a mean Dice score and sensitivity of 0.82 and 0.85 respectively, along with a mean root mean square error of 0.67 ± 0.35 mm between the thickness of predicted and ground truth portions of the bladder wall. Blueprint separable convolutions and adaptive attention-skip connections improve segmentation performance with fewer computations compared to respective standard counterparts. A comparative analysis demonstrates improvements between 2–20% in the dice score with respect to some but one of the existing networks and reductions in computational complexity by 94 – 96% with respect to all existing networks analyzed in this work. Therefore, the proposed method can be effective for accurate bladder wall segmentation and demonstrates potential for real-time application in clinical settings.

## I. Introduction

URODYNAMIC studies (UDS) are the most reliable methods available to assess bladder function, requiring invasive catheterization to estimate detrusor pressure [1]. During tests, bladder detrusor pressure is measured using two catheters placed in the bladder and one in the rectum or vagina during bladder filling. These measurements are utilized by healthcare providers to evaluate the bladder dysfunction [2]. However, the invasive nature of the UDS procedure can lead to patient discomfort and carries a risk of potential complications [3]. To address these challenges, our group introduced ultrasound bladder vibrometry (UBV), which is now referred to as “ultrasound urodynamic studies (US-UDS)” [4], a non-invasive approach to evaluate bladder dysfunction by estimating the detrusor pressure from ultrasound-generated Lamb waves in the bladder wall [5], [6], [7], [8], [9]. Fig. 1 illustrates a diagram of the UBV technique where an acoustic radiation force generated by an ultrasound push beam is exerted on the bladder wall, resulting in Lamb waves propagating along the anterior bladder wall. The group and phase velocity of these lamb waves can be estimated as demonstrated in [6]. The detrusor pressure can be estimated by using the phase velocity as described in [7]. This method requires precise segmentation of the anterior portion of the bladder wall prior to these evaluations.

Conventional segmentation methods are time-consuming and susceptible to manual variability in clinical settings [10]. Precise and reliable automated segmentation methods can address these limitations by providing an efficient clinical workflow and reducing the workload of radiologists and other healthcare providers [10]. Toward this end, deep learning- based methods are increasingly being utilized for automated segmentation of medical ultrasound images [11]. Among these, convolutional neural network (CNN)-based architectures, including fully convolutional and encoder-decoder-based networks, such as U-Nets, are particularly prominent, as they demonstrate remarkable segmentation performance [12], [13]. For ultrasound images, U-Net has been more widely used. Their performance is further enhanced by some recently proposed architectures, such as attention-based U-Nets and transformer-based networks [14], [15]. However, segmenting the anterior portion of the bladder wall in ultrasound images can be challenging and time consuming unless they are combined in a customized arrangement that effectively captures the relevant features of the target areas. This is because transformers fail to effectively capture the more relevant pixel locations and encoder-decoder-based structures may replicate less useful information from the encoder to the decoder [15]. In addition, there is a lack of clinical validation of these approaches, making it difficult to effectively segment the bladder wall.

Some studies focused on segmenting the entire bladder area in ultrasound images. For example, the researchers in [16] developed a portable ultrasound research system for postoperative bladder monitoring using Mask-R- CNN-based segmentation of the bladder area. Recently, researchers developed a memory-efficient low-compute deep learning network by quantifying the original U-Net, DeepLabv3+, and Panoptic feature pyramid networks for segmenting the bladder area [17]. The objectives of these works were the development of research systems for bladder monitoring in resource-restricted settings. Previously, our group used the U-Net architecture to segment the entire bladder wall, achieving an average dice score of 0.93 ± 0.04 [18]. Further, they estimated the thickness of the segmented bladder wall and reported an average root mean square error (RMSE) of 0.7 ± 0.21 mm with respect to that estimated manually. However, the overall computational and time complexity was high, and the performance was validated on a limited data set consisting only of healthy volunteers. Further, the focus of these works was towards extracting the entire bladder wall, while, the objective of our research is to extract the anterior portion of the bladder wall for UBV tests.

To address the aforementioned challenges, this work proposes a customized deep learning architecture designed for precise bladder wall segmentation in ultrasound bladder vibrometry, with potential for real-time operation. The proposed architecture inherits an encoder-decoder-like structure comprising computationally efficient blue-print-separable convolutional (BSConv-U) layers and hybrid adaptive attention mechanisms (*HA*) in the skip connections. In addition, we propose a boundary-aware loss function to effectively train the proposed network to handle class imbalance and missed boundary errors in the segmented portion. The proposed loss function merges the Dice and focal losses with an edge loss which is estimated as the binary cross-entropy loss between Sobel filter gradients of the predicted and ground-truth masks. The performance of the proposed architecture is validated in our recorded dataset. To ensure that the network captures informative features of the anterior bladder wall, we visualize feature maps via heatmaps, illustrating that the model focuses on anatomically relevant regions. Robust feature representations are critical for generalization across imaging conditions and datasets [19]. The main novelties of the proposed model from theory include: utilization of BSConv-U which performs computationally efficient feature extraction and optimizes the overall performance while minimizing the computational cost associated with standard convolutions; utilization of *HA* modules that selectively propagates spatially and channel-relevant features from the encoder to the decoder, enhancing representation of fine structures such as bladder-wall boundaries; introduction of boundary-aware loss encodes prior knowledge of region and boundary information to preserve the boundary and shape of anterior bladder wall. To the best of our knowledge, this is the first study to propose segmentation of the anterior portion of the bladder wall using blue-print separable convolutional layers and hybrid adaptive attention-based skip connections within an encoder-decoder-deep learning architecture. We summarize the main contributions of this work as follows.

- Adaptation of blueprint separable convolutional layers for computationally efficient extraction of the anterior portion of the bladder wall.
- Introduction of hybrid adaptive attention modules in skip connections for passing relevant details to the decoding stage.
- Introduction of a merged boundary-aware loss function during training to effectively capture the edges of the bladder wall.
- Implementation of some commonly existing deep learning architectures on our recorded dataset and performance analysis with respect to the proposed architecture.
- Demonstration of the potential real-time feasibility of the proposed method through a comprehensive computational complexity analysis, compared with existing models trained on our recorded dataset under similar operating conditions.

## II. Related Work on Deep Network Architectures for Medical Image Segmentation

We review relevant deep learning–based medical image segmentation frameworks including CNN variants, dynamic learning approaches, transformer-driven models, and Masked Autoencoder (MAE)-based methods to contextualize the design choices behind our proposed architecture.

### A. CNN-Based Medical Image Segmentation

CNNs are foundational for medical image segmentation due to their ability to learn hierarchical features from limited data. U-Net [13] introduced an encoder–decoder design with skip connections that preserves spatial information and enables accurate segmentation even with small datasets. Building on this, nnU-Net [20] offers a self-configuring framework that automatically adapts pre-processing, network architecture, and training strategies to different datasets, achieving state-of-the-art performance across diverse medical imaging tasks.

### B. Dynamic and Collaborative Learning Approaches

Dynamic learning approaches have become increasingly popular for scenarios with limited labeled data. Zeng et al. presented “Segment Together” [21], a collaborative framework that leverages consistency constraints across multiple predictions to improve segmentation accuracy and robustness under semi-supervised conditions. This approach is particularly useful for medical imaging tasks where expert annotations are costly and scarce.

### C. Transformer-Based Segmentation Models

Transformer-based architectures have been adapted to medical image segmentation to capture long-range dependencies that are difficult to model by CNNs. TransUNet [22] combines a Vision Transformer encoder with a U-Net–like decoder, integrating global context with precise localization. Variants such as Swin-UNet [23] further improve multiscale feature representation by employing hierarchical transformer blocks, enhancing the network’s ability to model fine-grained anatomical structures.

### D. MAE-Based Semi-Supervised Methods

Masked autoencoder (MAE)–based methods utilize reconstruction-guided pretraining to improve feature representation for segmentation tasks. “PICK: Predict and Mask for Semi-Supervised Medical Image Segmentation” [24] introduces a strategy where parts of the input are masked and the network learns to predict them, enhancing robustness to missing or ambiguous information. This approach improves boundary delineation and segmentation accuracy in semi-supervised settings, making it particularly useful for medical images with subtle structural details or limited labeled data.

The aforementioned architectures demonstrate strong performance in general medical image segmentation, however, they need to be specifically optimized for ultrasound-based bladder wall segmentation. Ultrasound data presents unique challenges such as speckle noise, partial visibility, and smooth wall curvature. Our proposed architecture addresses these issues by combining computationally efficient blueprint separable convolutions with hybrid adaptive attention modules in skip connections, enabling precise segmentation of the anterior bladder wall in UBV studies.

## III. Methodology

The proposed methodology includes a customized deep learning architecture fed with B-mode images of the bladder area as input along with the labeled images/masks as the ground truth during training.

### A. Study Design and Dataset Acquisition

In this work, we acquired images of the bladder region of 64 patient volunteers, including 18 female and 46 male volunteers, in a concurrent setting of UDS and UBV (US-UDS). The study was conducted under Mayo Clinic Institutional Review Board (IRB)-approved protocols: IRB# 11–001953 and IRB# 21–011734 and was HIPAA-compliant. Written informed consent, approved by the IRB, was obtained from each patient volunteer prior to participation. The mean age (standard deviation) and BMI of these patients were 67.04 (14.49) years and 27.88 (5.19) kg / m^2^, respectively. Each patient volunteer in this dataset had an established lower urinary tract pathology. The underlying clinical conditions included overactive bladder symptoms (urgency, frequency), bladder outlet obstruction/BPH-related symptoms (weak urinary stream, incomplete emptying, urinary retention), urinary incontinence and neurogenic or atonic bladder dysfunction. Urodynamic bladder status was categorized as normal compliant, non-compliant, detrusor overactive, or detrusor underactive. The acquisition of ultrasound image was performed during conventional catheter-based UDS, during cystometric filling. Once the cystometric filling was adequate for a clear view of the bladder, the UBV acquisition was started and a B-mode image of the bladder was saved every 5 seconds during filling. The imaging sequences were acquired at a center frequency of 3.57 MHz using the Vantage 256 ultrasound research system by Verasonics Inc, Kirkland, WA, USA with a curved array transducer, C5–2v, Verasonics Inc, Kirkland, WA, USA. In the UBV experiment, at the beginning of each sequence, a high-quality plane wave B-mode was acquired. It comprised 71 evenly spaced angle compounds (angular range of −17° to 17°). The number of B-mode images acquired from each subject ranged from 100 to 200, resulting in a total of 8592 images for all subjects. The images were resized to 512 × 512 pixels to be fed to the proposed deep learning network for segmentation.

### B. Labeling of the Bladder Wall for Ground Truth

For ground truth, the top of the bladder wall is labeled with multiple points along two edges, that is, upper and lower. The edge curves of the upper and lower wall are fitted with regression polynomial of order two as follows.

(1) $$\left\{\begin{array}{c} y_{1}=ax_{1}^{2}+bx_{1}+c+\theta \\ y_{2}=ax_{2}^{2}+bx_{2}+c-\theta \end{array}\right.$$

where $x_{1}$ and $y_{1}$ represents the horizontal and vertical axis coordinate of the upper bladder wall respectively; $x_{2}$ and $y_{2}$ horizontal and vertical axis coordinate of the lower bladder wall respectively. The parameter θ is half the thickness of the bladder wall. The two curves have same curvature and different intercepts. The ground truth mask is created by following steps.

1. The leftmost and rightmost labeled points are obtained as $\text{min}\left\{x_{1_{i}},\ldots x_{2_{j}},\ldots \right\}$ and $\text{max}\left\{x_{1_{i}},\ldots x_{2_{j}},\ldots \right\}$ respectively, where $i\in \left\{1,2,3,\ldots ,n_{t}\right\}$ and $j\in \left\{1,2,3,\ldots ,n_{b}\right\}$. $n_{t}$ and $n_{b}$ denote the total number of labeled points in upper and lower bladder wall respectively.
2. The horizontal axis coordinates of the leftmost point, $x_{\text{left}}$ and the rightmost point, $x_{\text{right}}$ are used in (1) to calculate the corresponding values of the vertical coordinates.
3. All points estimated from the previous step are connected to create the mask.

Fig. 2 shows an example B-mode image of bladder, with the labeled points on the upper and lower parts of the top of the bladder wall along with the corresponding ground truth mask obtained after joining these points.

To assess the accuracy and robustness of this annotation method, we performed an independent validation using manual delineations. A subset of 41 subjects was randomly selected, and manual contours of the anterior bladder wall were generated by an experienced radiologist. The fitted contours were then compared with these manual annotations. The average HD95 between the fitted and manual contours was 1.47 mm ± 0.5 mm, and the Dice score was 0.92 ± 0.05, indicating strong agreement and confirming the reliability of the proposed annotation approach.

### C. Proposed Deep Learning Architecture: BWS-Net

The proposed deep learning architecture comprises encoder-decoder-like network built using recently developed blueprint separable convolutional layers along with hybrid attention units in the skip connections, as shown in Fig. 3.

#### 1) Encoder:

The encoder utilizes a sequence of unconstrained blueprint separable convolutional layers followed by max-pooling layers as shown in Fig. 3. Blueprint separable convolutions were recently introduced by Hasse et al. in [25], and demonstrated to be more efficient as compared to the standard convolution. The standard convolutional layer applies constant N number of kernels/filters to a V channel input I of dimension V×A×B to obtain the convolved output O as:

(2) $$O_{j,:,:}=I*K^{\left(j\right)}$$

where O has dimension N×A×B and K denotes kernels/filters with j∈{1,…,N}, each of dimension V×F×F. The filter weights are optimized based on the backpropagation process during training and may assume arbitrary values. Fig. 4(a) illustrates this operation. It can be seen that the total number of multiplications is:

(3) A×B×F×F×N×V

The researchers in [25] analyzed that each V×F×F filter can be represented as a 2DF×F blueprint and V factors that ‘distribute’ the blueprint along the depth. Each filter $K^{\left(j\right)}$ can be redefined as $B^{\left(j\right)}$ and weight vector ${\tilde{w}}_{j}:w_{j,1},\ldots ,w_{j,V}\in R$ as:

(4) $$K_{v,:,:}^{\left(j\right)}=w_{j,v}\cdot B^{\left(j\right)},$$

where v∈{1,…,V}. Since there are N kernels, each with a blueprint and V weights, total V⋅N weights are used. An efficient formulation of (2) is further achieved by segregating each 3D convolution into an integral of V2D convolutions as follows [25].

(5) $$O_{j,:,:}=I*K^{\left(j\right)}=\sum_{v=1}^{V}\left(I_{v,:,:}*K_{v,:,:}^{\left(j\right)}\right)$$

Using (3) in (4), the following can be obtained:

(6) $$O_{j,:,:}=\sum_{v=1}^{V}\left(I_{v,:,:}*\left(w_{j,v}\cdot B^{\left(j\right)}\right)\right)$$

Since each blueprint is independent of the input channel and the weight vector is a scalar, the above equation can be re-organized into the following form [25]:

(7) $$O_{j,:,:}=\underset{O_{j,:,:}^{\prime }}{\underbrace{\left(\sum_{v=1}^{V}I_{v,:,:}\cdot w_{j,v}\right)}}*B^{\left(j\right)}$$

Further re-organizing the integration into convolution operation yields:

(8) $$O_{j,:,:}^{\prime }=I*{\tilde{w}}_{j}$$

(9) $$O_{j,:,:}=O_{j,:,:}^{\prime }*B^{\left(j\right)}$$

Equation (8) can be interpreted as a 1 × 1 convolution operation of the kernel weights with the input image I, which is a point-wise convolution [26]. The corresponding output of (8) is further convolved with the blueprints (kernels), each of dimension F×F, representing a depth-wise convolution [26]. This formulates the BSConv-U operation as a reversed depth-wise separable convolution. The two-step operation of BSConv-U is illustrated in Fig. 4(b). It can be seen that the total number of multiplications is A×B×N×V in (i) and A×B×F×F×N in (ii). Therefore, BSConv-U performs the total number of multiplications as:

(10) A×B×N×V+A×B×F×F×N

Dividing (3) by (10) yields the ratio of the total number of multiplications in BSConv-U to that in standard convolution operation as follows.

(11) $$\frac{1}{V}+\frac{1}{F^{2}}$$

It is apparent from the ratio that the computational complexity is reduced considerably by using the BSConv-U operation.

The above formulation shows that BSconv-U relies on intrafilter correlations, which helps in reducing time and parameters, while enhancing the capability of processing data with complex information. BSconv-U can efficiently separate noise from the input ultrasound images and enhance accuracy with the same dimension setting as standard convolution.

In this work, the encoder comprises nine BSConv-U layers in different blocks, with each block followed by maxpooling2D layers with a stride of two, as shown in Fig. 3. The first BSconv-U layer receives B-mode images of the bladder area as input, the corresponding output is fed to the second BSconv-U layer, and so on. The number of filters used in the encoding layers varies from 16 to 128, with each layer layer using a padding of 1 and Gaussian error linear unit (Gelu) activation. Layer normalization is used after every encoding layer, which performs better than the usual batch normalization in this task because of its dynamic mechanism based on each image.

#### 2) Decoder:

The decoder utilizes nine BSconv-U layers in different blocks, similar to the encoding layers. However, each block is followed by upsampling2D layers, each with a stride of two, to decode the output of the encoding part. The number of filters is similar to that of the encoding part. Unlike conventional encoder-decoder-based networks, the proposed network utilizes a hybrid attention (*HA*) module at the bottleneck, i.e. after the encoder unit, to provide more enriched features to the decoder unit. In addition, each decoding block receives the output of the corresponding encoding block after concatenation with a *HA*-based skip connection, which is proposed for the first time in this work. The *HA*-skip connections pass enhanced feature maps from the encoding stage, rather than passing them directly. The final layer of the decoder is an output layer which is a BSConv-U layer with a 1 × 1 kernel to predict the segmented mask.

#### 3) Adaptive Attention-Based Skip Connections:

The encoding layer outputs are concatenated to the corresponding decoding layers through skip connections in existing encoder-decoder-like architectures. In this work, we propose the use of adaptive attention (AA) mechanism prior to concatenation, which helps to extract an enriched feature map to be passed to the decoder. The adaptive attention mechanism was proposed by Chen et al. in [27]. However, this is the first work to inherit this mechanism for skip connections. AA utilized in this work includes the channel self-attention along with convolution using varying receptive fields. There are four AA modules, and each module receives the output of the BSConv-U block in the encoder as the input, shown in Fig. 3. The input is convolved in parallel with a 5 × 5 and a three-rate dilated 3 × 3 kernel to produce the outputs $A_{5}$ and $A_{D3}$, respectively. The different kernel sizes provide different receptive fields that capture variations and adaptations across different feature matrices [28]. The corresponding outputs are combined and down-sampled using global average pooling (GAP) as [28]:

(12) $$O_{G}=\text{GAP}\left(A_{5}⊕A_{D3}\right)$$

The feature map $O_{G}$ is passed to a dense layer to generate $O_{D1}$, which is further passed to gaussian error linear unit (GELU) activation to obtain $O_{D1^{\prime }}$ as:

(13) $$O_{D1^{\prime }}=\sigma_{G}\left(\text{BN}\left(W_{\text{De}}\cdot O_{D1}\right)\right)$$

where $\sigma_{G}()$ denotes the GELU activation, BN denotes batch normalization, and $W_{\text{De}}$ denotes the weight vector of the dense layer. The corresponding output is again fed to a dense layer to obtain $O_{D2}$, which is passed through a sigmoid activation to obtain the channel attention weights for scaling the initial inputs $A_{5}$ and $A_{D3}$ [28]. Finally, these weighted features are combined to produce the adaptive attention output $O_{\text{AA}}$ [28]. It helps to effectively combine features from different receptive fields. These operations are represented as:

(14) $$O_{\text{AA}}=\alpha ⊗A_{5}+(1-\alpha )⊗A_{D3}$$

where $\alpha =\sigma_{s}\left(W_{D}\cdot O_{D2}\right)$. Finally, the attention output is concatenated to the decoder. This skip connection helps to effectively refine the information with relevant features before passing it to the decoder.

The proposed network was trained in the PyTorch deep learning framework in Python. All experiments were performed on a workstation equipped with an NVIDIA L40 GPU, featuring 48 GB GDDR6 ECC memory, 864 GB/s memory bandwidth, and PCIe Gen4 × 16 connectivity. The system CPU was an Intel Xeon Silver processor (2.1 GHz), with 128 GB RAM. Under this deployment setting, the proposed model achieved an average inference time of 4.2 ms per 512 × 512 ultrasound frame, corresponding to approximately 238 frames per second, which exceeds typical ultrasound real-time requirements (10–30 FPS), indicating potential for real-time feasibility. The GPU memory footprint during inference was approximately 1.2 GB, while system RAM usage was about 9 GB. The average training time on the L40 GPU was approximately 6 minutes per epoch, with a peak GPU memory usage of about 10 GB during training. For training, we used the B-mode images of 80% of the subjects. The data of the remaining 20% subjects were further split into 10% each to test and validate the proposed network, respectively. The model was trained using a batch size of 8. The initial learning rate was set to 0.001 and decayed to 0.0001 during training. Data augmentation was applied to each training sample using random rotations, horizontal flips, and zooming, with an augmentation factor of 3, effectively tripling the size of the training dataset. The model was trained for up to 300 epochs using Adam optimizer, and training was stopped early if the validation loss did not improve for a set number of epochs. Dice score was used as the training metric. A customized loss function is introduced in this work, which is a boundary-aware loss function derived from the combination of Dice loss [29], focal loss [31], and the boundary error of the segmentation mask, to handle class imbalance and missed boundaries. Let $P\in [0,1{]}^{N}$ be the predicted probability map, and $G\in \{0,1{\}}^{N}$ be the binary ground truth mask. The BFD Loss is defined as:

(15) $${ℒ}_{\text{BFD}}=\lambda_{1}\cdot {ℒ}_{\text{Dice}}+\lambda_{2}\cdot {ℒ}_{\text{Focal}}+\lambda_{3}\cdot {ℒ}_{\text{Boundary}}$$

where $\lambda_{1}$, $\lambda_{2}$, and $\lambda_{3}$ are the attention weights for each term. To enforce sharp boundaries, we introduce a boundary-aware loss based on the Sobel gradient operator and estimate it as follows.

(16) $${ℒ}_{\text{Edge}}=\text{BCE}(\text{Sobel}(P),\text{Sobel}(G))$$

where BCE(·) is the binary cross-entropy between the predicted and ground truth boundary masks. Based on experimental analysis, we use $\lambda_{1}=1.0$, $\lambda_{2}=1.0$, and $\lambda_{3}=0.5$. All notations used in methodology are described in Table IV.

The network architecture and hyper-parameters were selected according to the configuration specified in the dictionary “search_space”. The search explored key parameters including learning rate, batch size, number of convolutional filters, network depth, dropout rate, optimizer type, and the weighting of components in the boundary-aware loss. The final configuration was selected based on the highest Dice score on the validation set, ensuring an optimal trade-off between segmentation accuracy, stability, and computational efficiency.

## IV. Results

The proposed network was trained and validated using the acquired dataset. Different segmentation metrics were computed using a five fold cross-validation scheme. These include Dice score (*Dice*), sensitivity (*Se*), precision (*Pr*), F-1 score (*F*1), Mathew’s correlation coefficient (*MCC*), Jaccard index (*JI*), and area under the receiver operating characteristics curve (*AUC*) [29]. In addition, we estimated the 95^*th*^ percentile Hausdorff distance (*HD*95) [30] to further assess boundary accuracy.

### A. Performance Metrics

We evaluated the performance of the proposed network using a five-fold cross-validation scheme. In each fold, we divide the data set into 80% for training, 10% for internal validation (during training), and the remaining 10% for external validation/testing. In this way, we tested the proposed network on all 8592 images while ensuring that there is no overlap between the train and the test subjects in the folds. We computed the average values of the segmentation metrics across all folds as follows. *Dice* of 0.82 ± 0.11 (mean ± standard deviation), *MCC* of 0.82 ± 0.09, *F* – 1 of 0.82±0.12, *Pr* of 0.84 ± 0.06, *Se* of 0.88 ± 0.09, *JI* of 0.74 ± 0.09, and *HD*95 of 3.5 ± 0.35 mm were achieved. These results demonstrate the effectiveness of the proposed network in detecting the anterior bladder wall. We also present Fig. 5 to demonstrate the average segmentation metrics achieved for all images. It illustrates the median values along with their range in the data. For example, the values of *Dice* are between 0.58 – 0.94, with the lower and upper quartiles between 0.74 – 0.85, along with a median value of 0.79. This distribution shows that most of the images are segmented with high dice scores. In addition, it can be observed that there are only eleven outliers, which correspond to approximately 1.2% of the images, indicating the potential generalizability of the proposed network. Similar interpretations can be drawn for the other metrics. The *JI* is low, which indicates small over- or under-segmentation scenarios, however, the overall performance is reliable. The sensitivity can be observed to be higher among other metrics, indicating that the proposed network is capable of accurately capturing the anterior portion of the bladder wall while minimizing false negatives. Furthermore, the *HD*95 exhibited consistently low values, with lower and upper quartiles of 0.56 mm and 7.96 mm, respectively, and a median of 2.67 mm. These results highlight the effectiveness of the boundary-aware loss in enhancing segmentation boundary fidelity.

To further analyze the performance of the proposed network, we estimate the thickness of the segmented anterior portion of the bladder wall. Fig. 6 shows the distribution of the mean thickness values obtained after using the predicted segmented masks and that using the ground truth masks for the test images. The values follow a similar distribution, with a slight decrease observed for the values obtained from predicted masks (overall mean: 5.39 ± 0.66 mm) compared to that obtained from the ground truth masks (overall mean: 5.64 ± 1.21 mm). We also measured the root mean square error (RMSE) between the thickness values obtained from the predicted and ground truth cases, which was 0.67 mm ± 0.35 mm. These results demonstrate a close proximity between the mean thicknesses of the anterior portions of the bladder walls extracted from the proposed network and the truth of the ground. Finally, this indicates the effectiveness of the proposed network while automating the complete process of thickness estimation.

### B. Ablation Study

To further analyze the effectiveness of the proposed network architecture, we performed an ablation analysis. Initially, we removed the proposed AA-skip connections and replaced them with standard skip connections. Next, we removed the BS-Conv-U layers and replaced them with the standard convolutional layers, while restoring back the AA-skip connections. For both cases, we estimate the corresponding mean segmentation metrics for the test set, as presented in Fig. 7. A substantial decrease can be observed in the overall performance metrics of the proposed network without the BS-Conv-U layers. For example, the mean *Dice* score and *MCC* drop from 0.82 to 0.77, which corresponds to approximately 6% decrease. The mean *F* – 1 score, *Se*, and *JI* decrease considerably from 0.83, 0.85, and 0.74 respectively, to 0.77 (7%), 0.76 (10%), and 0.68 (8%), respectively. Similarly, removing the AA-skip connections also reduces overall performance; however, the reduction is slightly less than that observed without BS-Conv-U layers. Similar performance degradation is observed in terms of HD95, which increases with these ablation cases. Finally, these results indicate the effectiveness of blueprint separable convolutional layers as well as AA-skip connections in the proposed network.

To evaluate the effect of the boundary-aware loss in our network, we conducted an ablation study by systematically varying the attention weights of the three loss terms, i.e., $\lambda_{1}$ (Dice, $\lambda_{2}$ (Focal), and $\lambda_{3}$ (Boundary). The values of weights were selected based on existing related studies [32], [33] where Dice- and Focal-based terms typically require weights close to unity (approximate range 0.5 − −1.5), while boundary-aware terms generally need smaller values (< 1) to avoid overestimating the boundaries. Table I demonstrates the means (standard deviations: std.) of performance metrics with respect to the different loss weights. We observed progressive reduction in HD95 with increasing $\lambda_{3}$, indicating the role of the proposed boundary term in improving contour localization. However, larger $\lambda_{3}$ value (0.75) slightly reduced Dice, indicating an over-estimation of edges. Further, reducing $\lambda_{1}$ and $\lambda_{2}$ decreased the overall Dice score and other metrics, while increasing them resulted in slight increase in HD95 and decrease in other metrics. Finally, the optimal performance was achieved with the selected combination of $\left(\lambda_{1},\lambda_{2},\lambda_{3}\right)$ as (1,1,0.5) which provided the best balance between overall overlap and the boundary-level accuracy.

### C. Interpretability of the Proposed Network

To assess the interpretability of the proposed network and its adaptive attention (AA)-skip mechanism, we generated the intermediate feature representations along with attention maps across the encoder–decoder pathway, as shown in Fig. 8. For each resolution level, we first display the raw encoder feature maps (top to bottom), which capture increasingly abstract structural information. Adjacent to these, we show the attention-refined feature maps generated by the AA block at the same level (top to bottom), and finally the decoder feature maps (bottom to top) that integrate these attention-skip maps. It can be observed that at the lower-resolution stages (Attention-1 and Attention-2), the refined attention maps show clearer localization (higher intensities) around the curved contour of the anterior bladder wall compared to the corresponding encoder outputs, indicating that the AA mechanism begins filtering irrelevant information. As the network depth increases, the AA module consistently suppresses background speckle and irrelevant tissue textures while enhancing relevant structural patterns associated with the anterior bladder wall boundary (Attention-3,4). This progressive refinement demonstrates the AA module’s ability to guide feature extraction towards anatomically relevant region, i.e., the anterior bladder wall. These visualizations demonstrate the contribution of AA-skip connections towards relevant feature selection and complement the quantitative improvements observed in the ablation study. These refined representations guide the decoder reconstruction process by providing spatially focused outputs. The AA-refined representations support the decoder in more accurately propagating boundary-related information through the upsampling path. Overall, the results illustrate how the AA module enhances encoder features and enables the decoder to reconstruct a more precise anterior bladder wall segmentation.

### D. Comparative Analysis With Respect to Some Existing Medical Image Segmentation Methods

We performed a comparative evaluation of our proposed network with respect to some state-of-the-art deep learning models for medical image segmentation tasks. For this purpose, we implemented these models and trained and tested them in our data set to segment the anterior portion of the bladder wall. These include U-Net [13], TransU-Net [22], ResU-Net [34], R2U-Net [35], AttentionU-Net [36], SwinUNet [23], Dual Self Distillation U-Net [37], and nnU-Net [20]. We computed all segmentation metrics, along with *HD*95 and computational complexity in terms of floating-point operations (*FLOPs*). Table II shows the corresponding metrics along with the FLOPs for all networks and the proposed network. It can be observed that among the existing models, nnU-Net demonstrates the best performance while utilizing fewer FLOPs, followed by attention-U-Net, SwinUNet, Dual Self Distillation U-Net and ResU-Net, with attention-U-Net utilizing a lower number of FLOPs. The performance of U-Net is almost comparable with these two networks, while its FLOPs are considerably higher than that of the attention-U-Net. The performance of TransU-Net is considerably lower in terms of *Dice*, *MCC* and *F* – 1, while the other metrics are comparable with U-Net, along with lower *FLOPs*. Among existing networks, R2U-Net performs the worst, although it requires fewer *FLOPs* than all existing networks except nnUNet. Finally, it can be observed that the proposed network outperforms the existing models U-Net, TransU-Net, ResU-Net, R2-U-Net, Attention-U-Net, SwinUNet and Self Distillation U-Net in terms of all metrics. In addition, it uses a substantially low number of *FLOPs* (13.85) compared to that utilized by these networks (219.88 – 401.52), indicating a considerable reduction by a factor of around 16 – 29 respectively, in the computational complexity of the proposed network with respect to these networks. Although nnU-Net shows relatively higher *Dice*, *MCC*, and *F* – 1, and comparable *JI* and *HD95*, the sensitivity and precision are slightly lower. Furthermore, it requires considerably higher FLOPs, which indicates an increase by a factor of about 11in its overall computational complexity with respect to the proposed method, indicating a trade-off. Therefore, the proposed method provides robust segmentation performance with lower computational complexity, making it particularly suitable for clinical bladder wall analysis where accurate boundary delineation and efficiency are critical.

We performed a statistical analysis of the proposed method with the state-of-the-art (baseline) models by conducting paired t-tests and Wilcoxon signed-rank tests on the per-fold Dice scores. Table III shows the corresponding p-values for both tests. The proposed method demonstrated statistically significant improvements over U-Net, R2U-Net, TransU-Net, and Attention U-Net, with p-values below 0.05 in both the paired t-test and Wilcoxon tests. Notably, the differences compared to R2U-Net and TransU-Net were highly significant (e.g., $p=9\times 10^{-6}$ for R2U-Net), indicating consistent superiority across folds. Comparisons with ResU-Net, SwinU-Net, and the Dual Self-Distillation U-Net demonstrated borderline significance in the paired t-test (p=0.040--0.045), while the Wilcoxon test resulted in p-values slightly above the 0.05 threshold, suggesting marginal improvements. As expected, no statistically significant difference was observed between the proposed method and nnU-Net (paired t-test: p=0.310; Wilcoxon: p=0.340), reflecting their comparable Dice performance. Overall, these results confirm that the proposed method achieves significant improvements over these architectures while maintaining competitive accuracy relative to the strong nnU-Net baseline, with substantially lower computational cost.

We present Fig. 9 to demonstrate the segmented masks of the bladder wall predicted by the proposed network and the existing networks for four different images. For all images, it can be observed that among the existing networks, TransU-Net fails to capture the mask, R2U-Net captures the mask with an improper shape with substantial false positives, while other methods, such as AttentionUNet, Self-Distillation U-Net, Swin-U-Net, yield competitive results but sometimes miss finer boundary details or show minor artifacts. nnUNet captures these details and produces masks close to the ground truth. However, the proposed network captures the fine details along with the boundaries of the bladder wall with slightly more precision with respect to the ground truth masks, thereby outperforming these models. Finally, these results demonstrate the ability of the proposed network to effectively segment the anterior portion of the bladder wall with low computational complexity. This further indicates that the proposed network architecture is customized to segment the anterior portion of the bladder wall.

## V. Discussion

This work presents a novel and computationally efficient deep learning framework, BWS-Net, designed specifically for the segmentation of the anterior bladder wall in ultrasound images. BWS-Net combines two lightweight architectural innovations in the context of anterior bladder wall segmentation: Blueprint Separable Convolutions (BSConv-U) and Adaptive Attention (AA) skip connections. BSConv-U substantially reduces computational cost while maintaining feature learning capability, indicating potential feasibility for real-time applications. AA connections further enhance performance by dynamically weighting encoder-decoder features, preserving the spatial detail features, thereby, improving segmentation of the anterior bladder wall. This combination enables BWSNet to achieve high accuracy with significantly reduced computational complexity, making it suitable for deployment in point-of-care or embedded ultrasound systems.

The performance evaluation of the proposed network demonstrates its effectiveness in accurately detecting the anterior bladder wall. The results highlight the model’s robustness across images of different patients, showcasing its effective generalization ability despite variations in data distribution. Thickness estimation of the segmented bladder wall further confirms the model’s accuracy, showing close agreement with ground truth measurement-an essential factor for subsequent UBV-based detrusor pressure estimation. Overall, the evaluation demonstrates that the proposed network reliably segments the anterior portion of bladder wall while automating thickness estimation, underscoring its potential for clinical applications.

The ablation study further demonstrates that both the BSConv-U layers and the AA-based skip connections independently contribute to the performance improvement. The removal of either component results in a noticeable decline in segmentation metrics, highlighting the effectiveness of these architectural components.

The comparative evaluation with respect to state-of-the-art approaches such as U-Net, TransU-Net, SwinU-Net, Dual Self Distillation-U-Net, and nnU-Net, demonstrates that BWSNet achieves superior performance and competitive Dice scores while utilizing substantially less computations. One of the most significant findings of this work is the drastic reduction in computational complexity. BWS-Net achieves 94–96% fewer FLOPs compared to conventional networks such as U-Net, ResU-Net, SwinU-Net, Dual Self Distillation-U-Net and Attention U-Net, while maintaining and in several cases, exceeding segmentation accuracy. This makes BWS-Net highly suitable for efficient deployment in clinical environments as also indicated by the previously reported quantitative metrics and inference time, particularly during procedures like ultrasound bladder vibrometry (UBV), where timely segmentation is important, and indicates potential for real-time operation. Notably, while models like TransU-Net and nnU-Net rely on transformer modules or extensive architectural tuning, BWSNet achieves comparable or better segmentation performance without sacrificing efficiency, demonstrating a favorable trade-off and potential suitability for real-world deployment. Its improved sensitivity and precision is particularly important for detecting slight bladder wall thinning, a key indicator of non-compliant bladders. While nnU-Net slightly outperforms BWS-Net in some segmentation metrics, it performs at 11-fold increase in FLOPs, which limits its applicability in time-sensitive or resource-constrained clinical settings.

The performance advantages of BWS-Net over baseline architectures can be attributed to the combination of three key architectural components. First, the blueprint-separable convolution (BSConv-U) layers minimize redundant computations while preserving the feature-extraction capability of standard convolutions. Second, the adaptive attention modules enhance the skip connections by selectively emphasizing spatially relevant features of the anterior bladder wall. The qualitative heatmap visualizations further support this, showing that the AA modules consistently focus on the anterior wall while suppressing irrelevant background structures. These refined features allow the decoder to emphasize important spatial regions, improving the accuracy of the predicted bladder-wall boundaries. Third, the boundary-aware loss integrates region-based and edge-based losses to explicitly penalize boundary inaccuracies, addressing common errors such as leakage or missing edges seen in standard segmentation losses. Finally, these components enhance feature discrimination, improve boundary precision, and help the model maintain a strong balance between accuracy and computational efficiency, as observed across all comparisons.

Clinically, BWSNet has the potential to improve the accessibility and efficiency of bladder compliance assessment. Current gold-standard methods rely on invasive pressure-based urodynamics, which are uncomfortable for patients and resource-intensive for clinics. By enabling potentially real-time, non-invasive estimation of anterior bladder wall thickness from standard ultrasound, BWSNet supports faster, more patient-friendly evaluation of lower urinary tract dysfunction. Its low computational requirements make it feasible for integration into portable ultrasound systems or point-of-care ultrasound workflows, potentially expanding access to bladder diagnostics in resource-limited settings.

Despite these promising results, there are certain limitations of this work. Firstly, external public datasets for anterior bladder-wall segmentation in UBV or conventional ultrasound are currently unavailable. As a result, the evaluation was conducted on our institutionally acquired dataset. Although this limits direct cross-dataset generalization testing, the dataset comprises a wide range of subjects and real-world imaging conditions, enabling a meaningful assessment of the proposed model. Future work will focus on curating and releasing a larger multi-center dataset to facilitate better generalizability. Secondly, the model is trained exclusively on 2D B-mode ultrasound, which lacks volumetric and temporal information. The integration with 3D ultrasound data can fully capture bladder wall dynamics, particularly during vibrometry in future extensions of this work. Integration with other modalities, such as Doppler or elastography, may enhance both spatial context and segmentation accuracy in future extensions of this work. Finally, deployment on embedded devices and validation in clinical workflows will be essential to realize the full potential of BWS-Net in replacing invasive urodynamic procedures.

## VI. Conclusion

In this work, we propose a customized deep learning architecture to segment the anterior portion of the bladder wall using ultrasound imaging. Performance evaluation on our recorded dataset demonstrates that the proposed network can effectively extract the anterior portion of the bladder wall. The thickness analysis of the predicted versus ground truth anterior portion of the bladder wall further demonstrates the adeptness of the proposed network. The ablation analysis indicates that utilizing the blueprint separable convolutional layers and adaptive attention-based skip connections considerably improves the overall segmentation performance. Further comparative analysis demonstrates that the proposed network outperforms many of the existing deep learning networks for medical image segmentation while utilizing substantially less number of floating point operations. Finally, the analysis presented in this work indicates that the proposed network is customized to segment the anterior portion of the bladder wall and exhibits low computational complexity, demonstrating its potential for real-time operation. In particular, our proposed network can facilitate the non-invasive assessment of bladder dysfunction.

## Figures and Tables

**Fig. 1.**
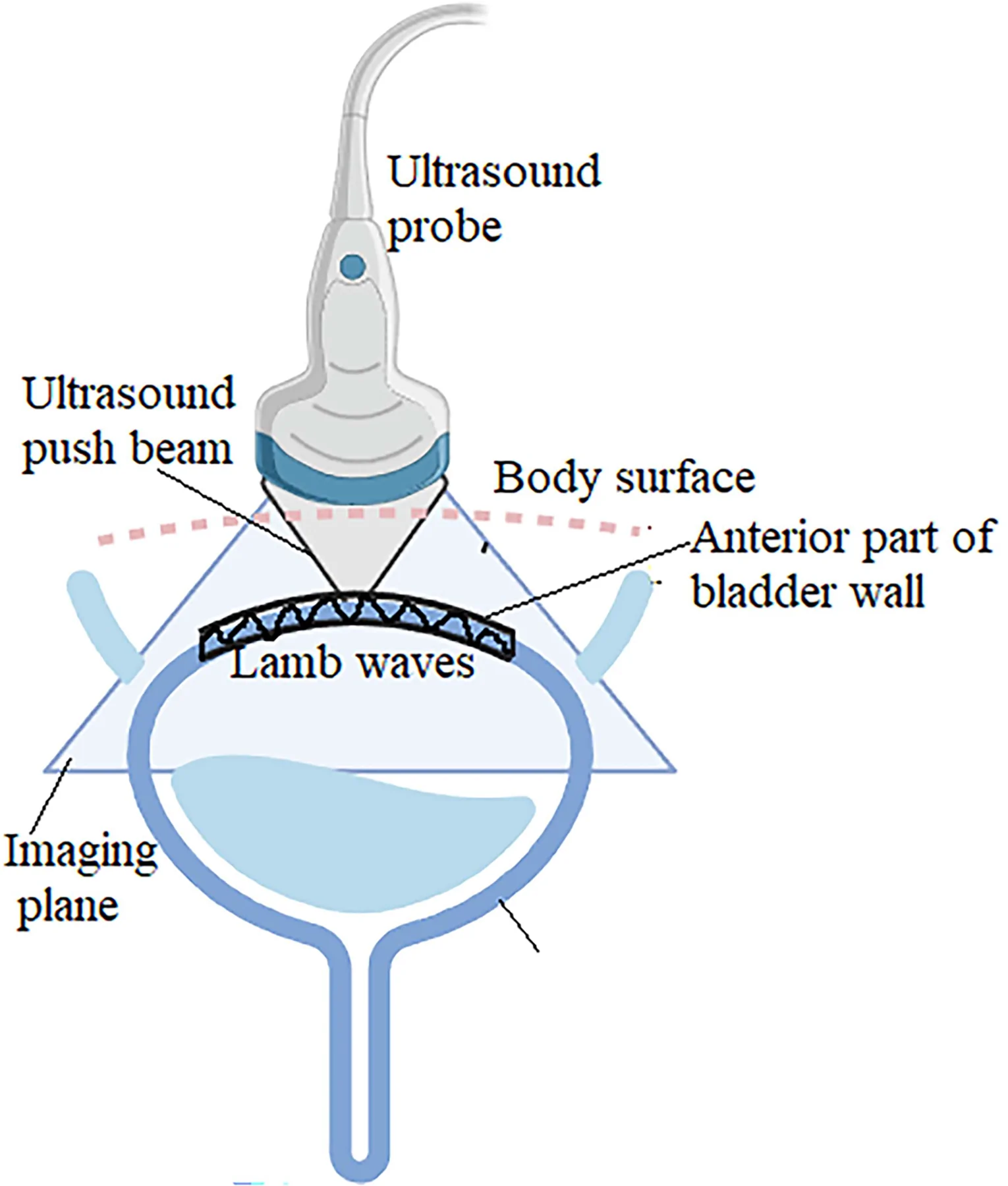
Ultrasound Bladder Vibrometry (UBV) system for studying bladder compliance.

**Fig. 2.**
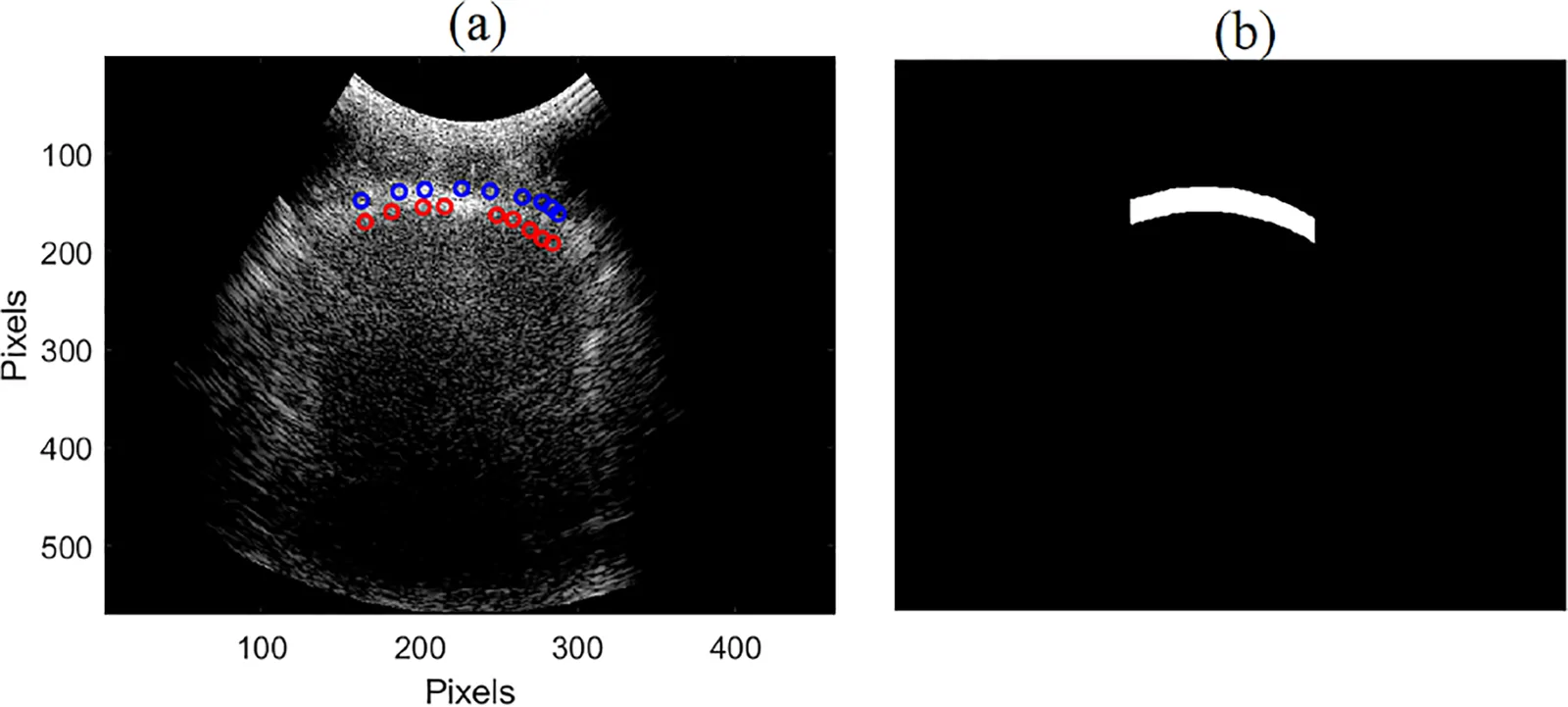
Labeling of brightness mode (B-mode) images for ground truth: (a) B-mode image with the points marked across the bladder wall curve (upper wall in blue and lower wall in red); (b) The segmented mask obtained after connecting the points.

**Fig. 3.**
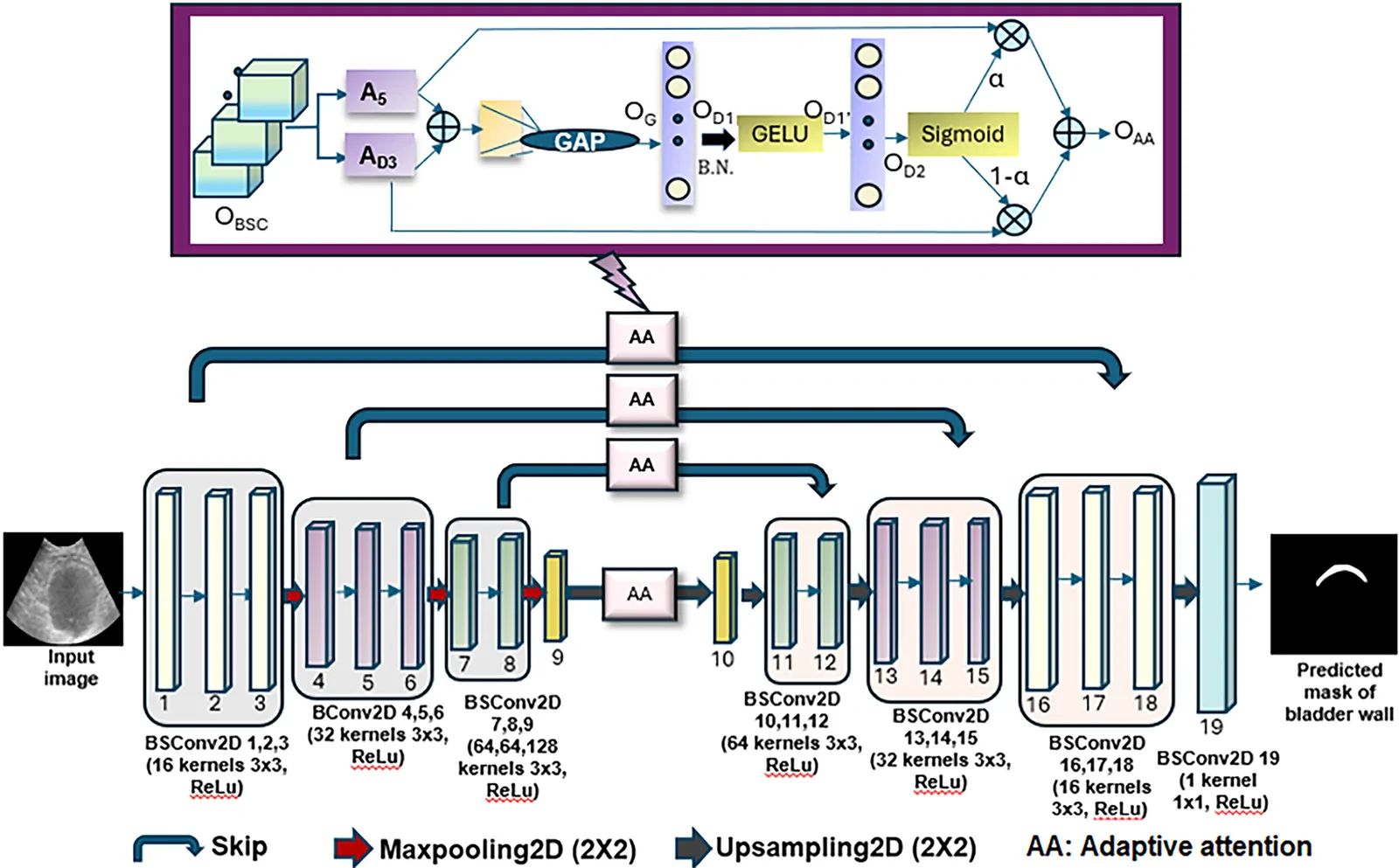
Proposed bladder wall segmentation network (BWS-Net).

**Fig. 4.**
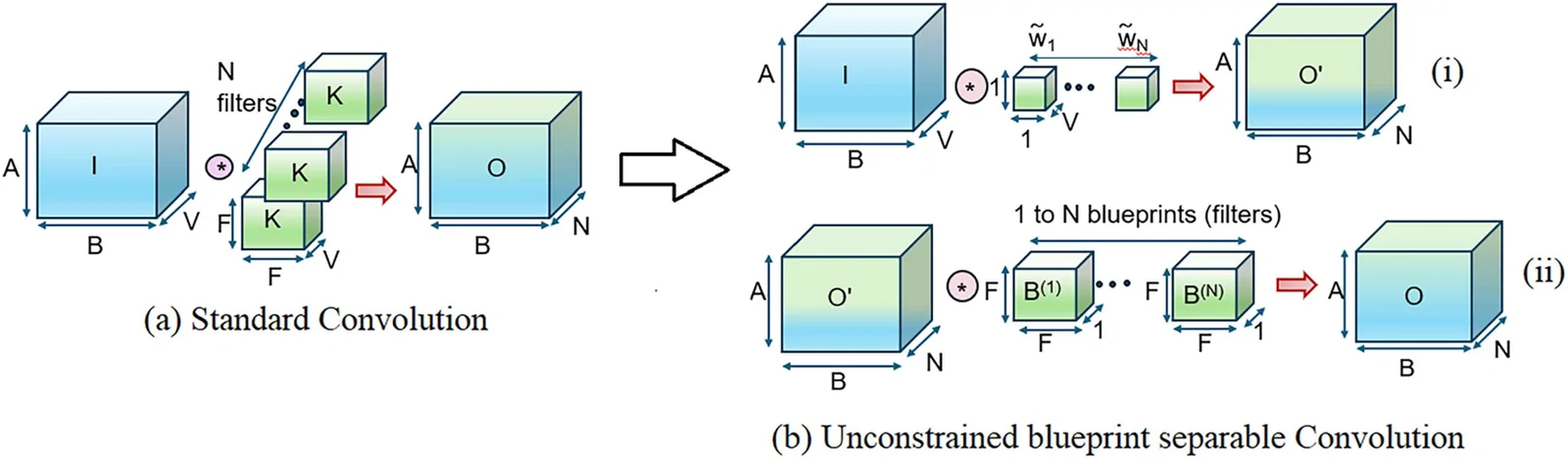
Illustration of standard and unconstrained blueprint separable convolution operation.

**Fig. 5.**
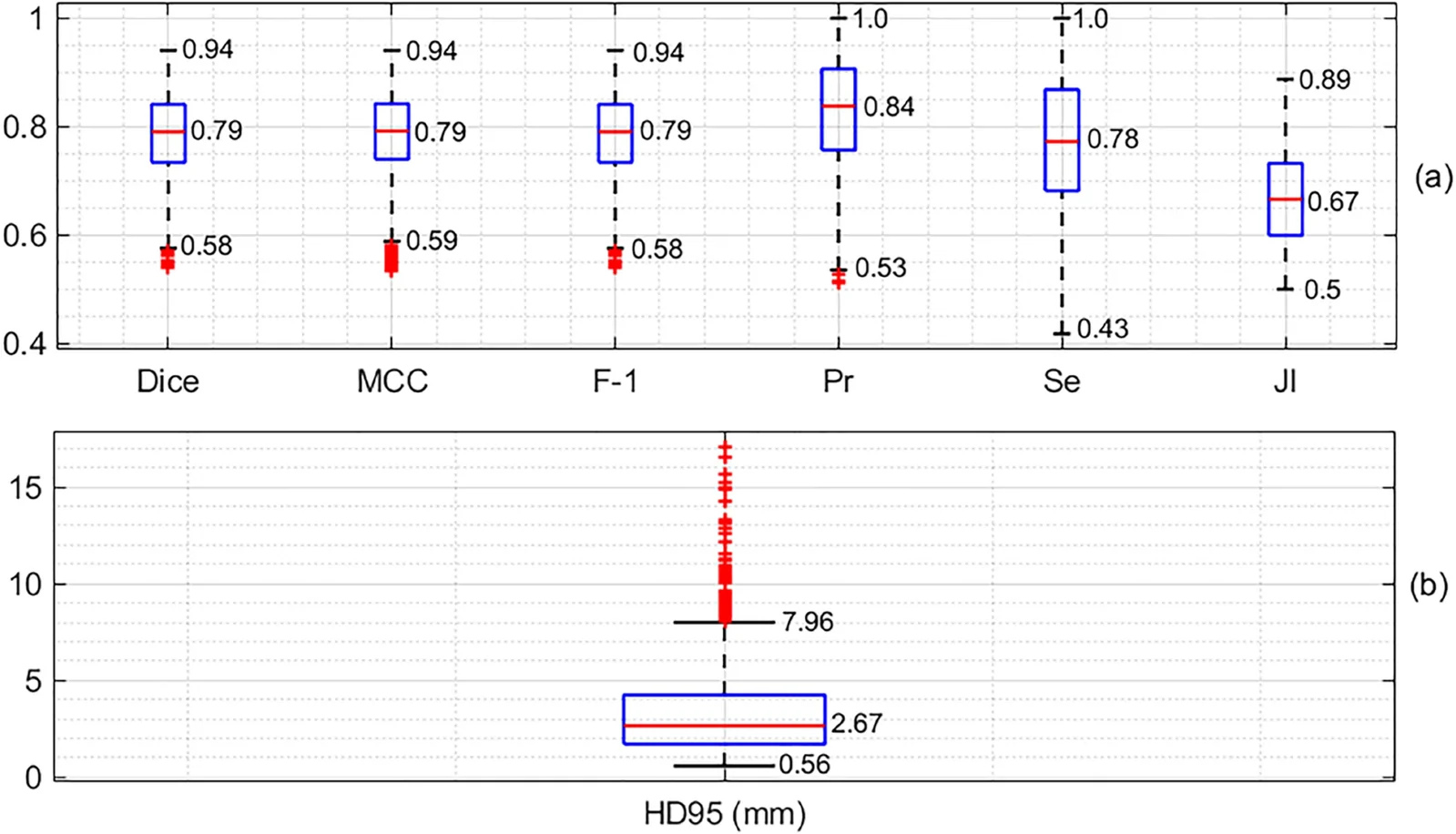
Distribution of the segmentation metrics achieved for all images.

**Fig. 6.**
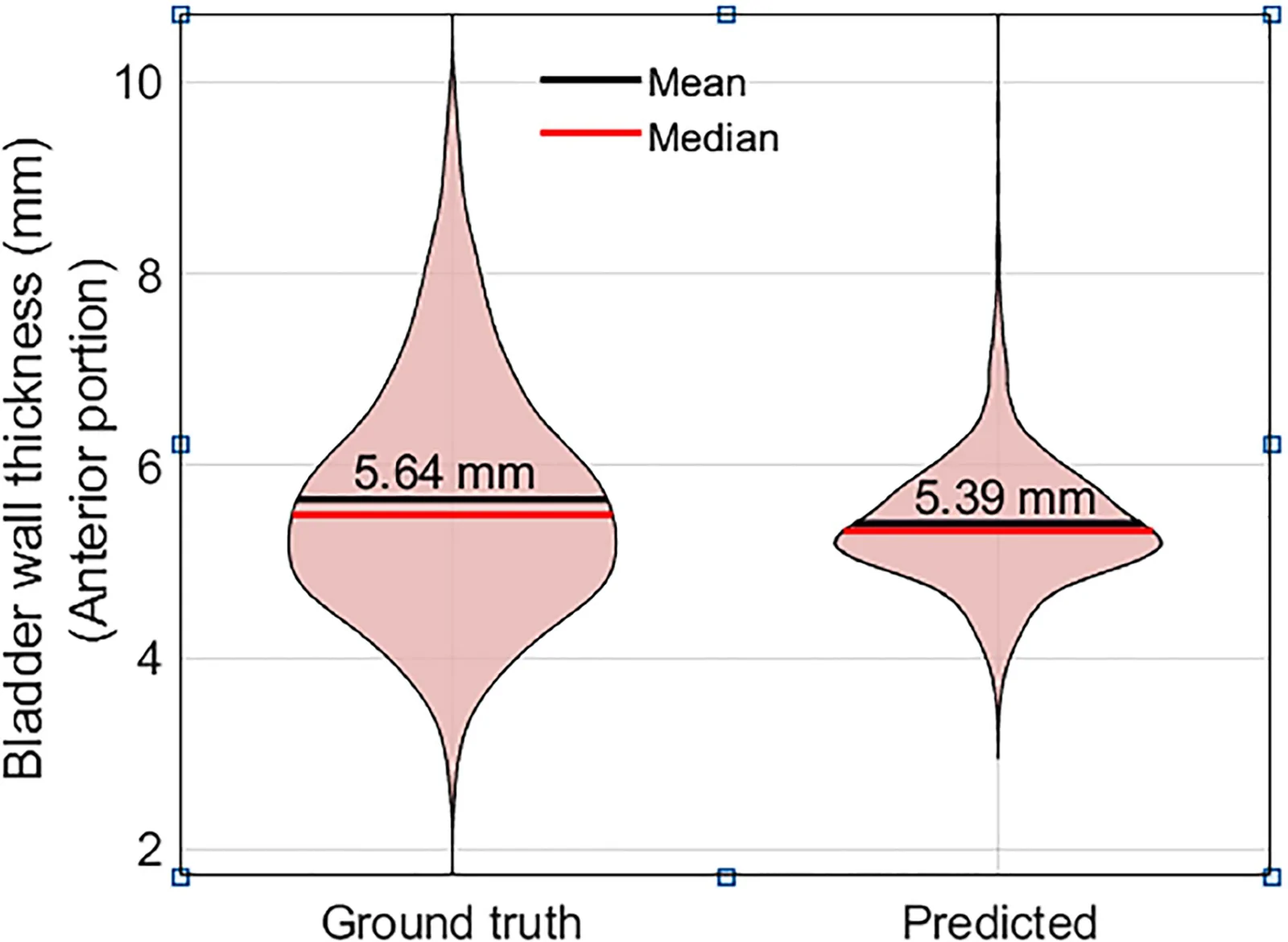
Illustrates the distribution of thicknesses of anterior portions of the bladder walls segmented by the proposed network versus ground truth, for the test images.

**Fig. 7.**
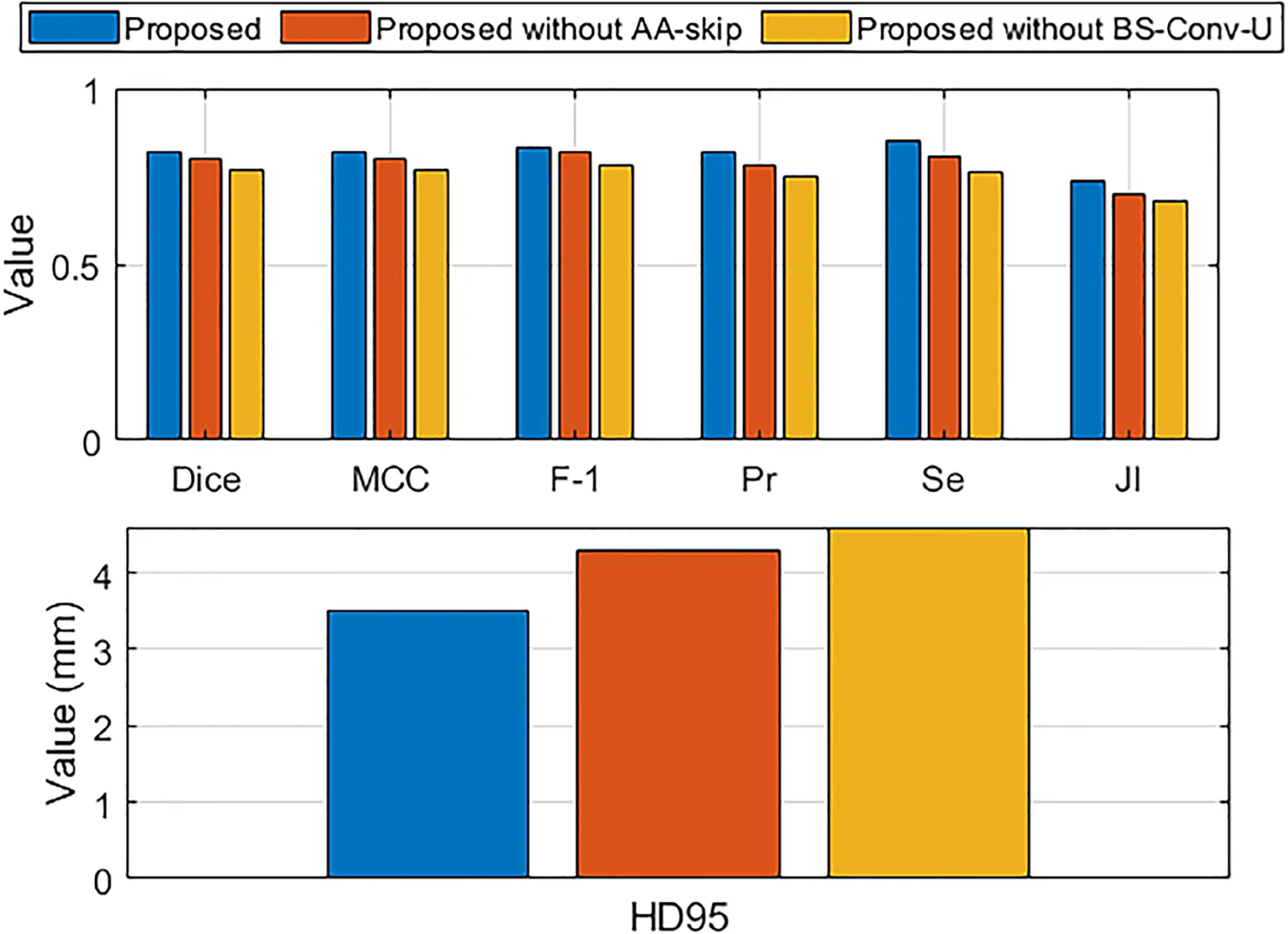
Test segmentation metrics (mean) for different ablation cases and the proposed network.

**Fig. 8.**
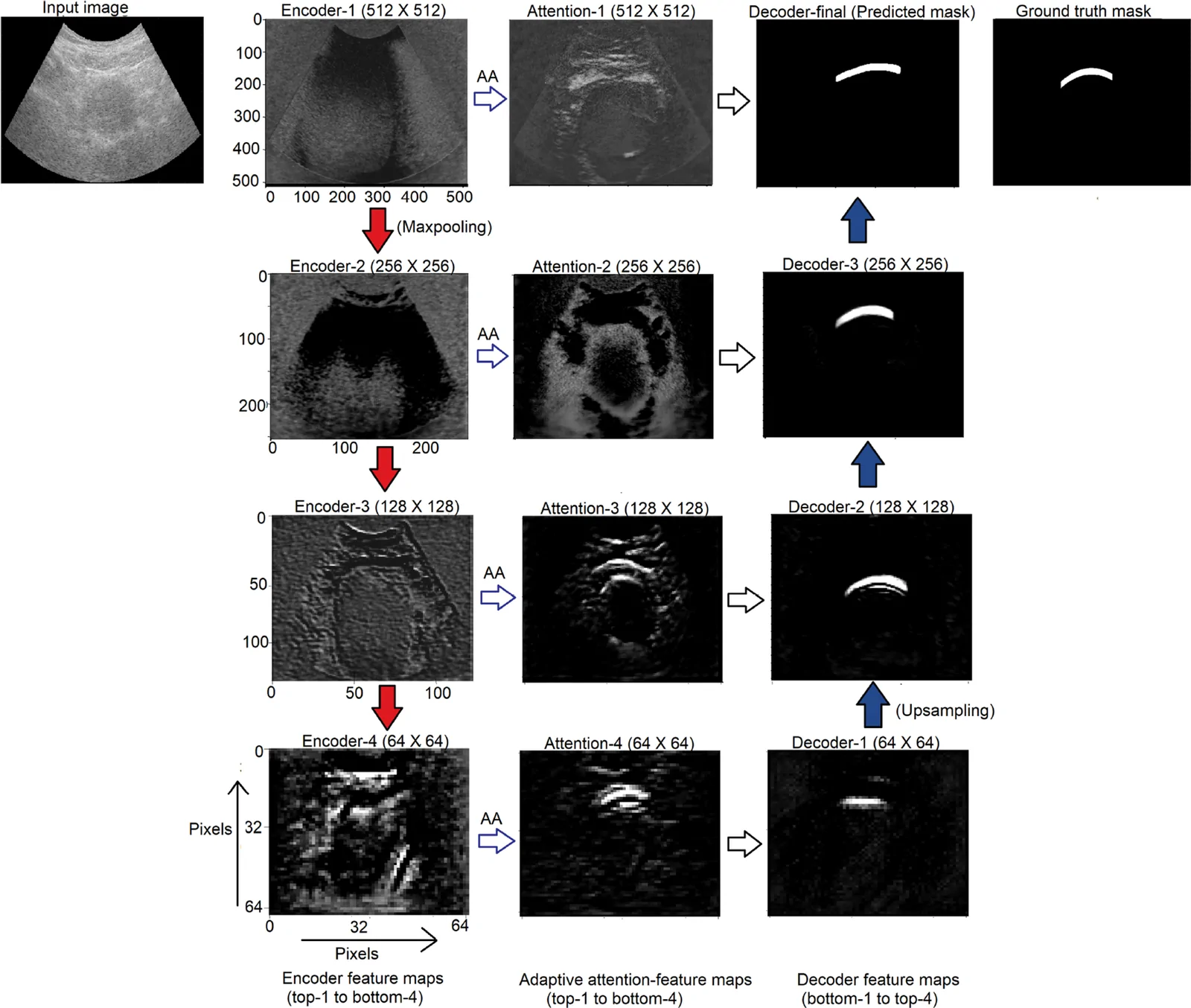
Feature maps generated by the proposed model for an input image (top left). For each encoder stage (left column), the corresponding adaptive attention maps are shown (middle column), followed by decoder feature maps produced during upsampling (right column). The final predicted segmentation mask is compared with the ground truth. The visualization highlights how attention-guided feature refinement enhances boundary localization.

**Fig. 9.**
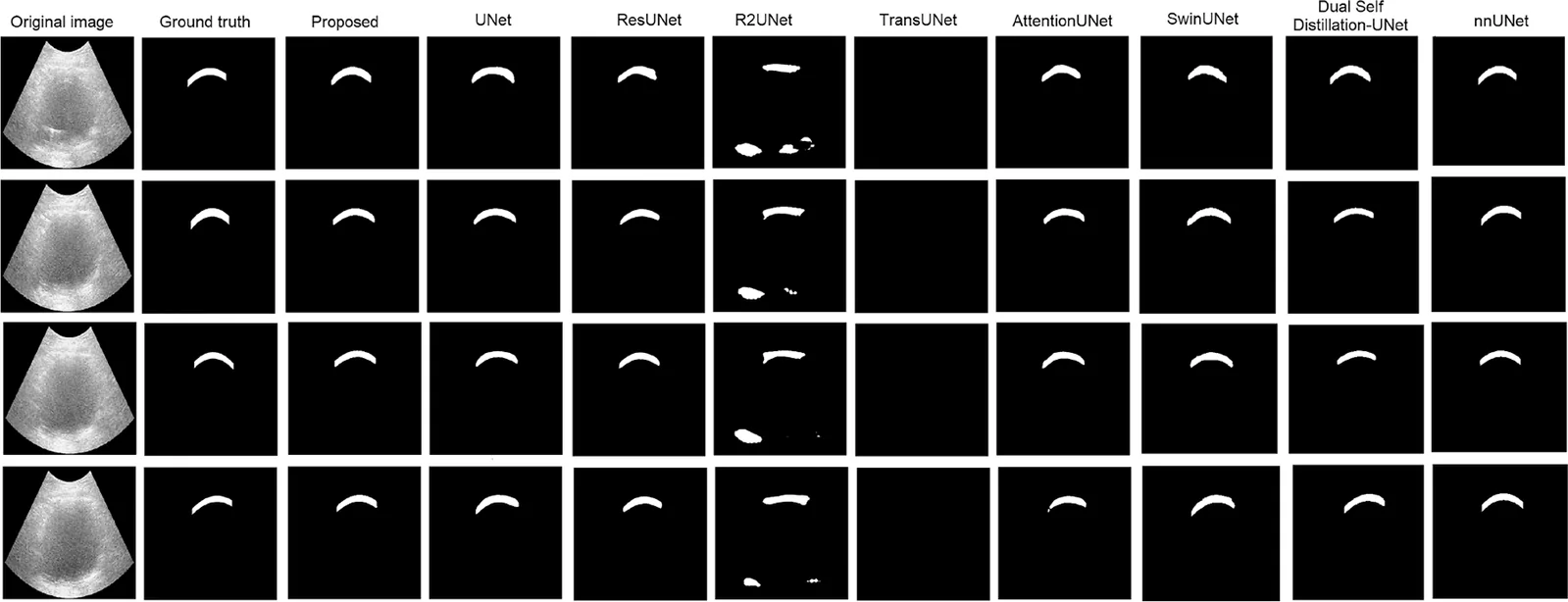
Qualitative comparison: Illustrates four different B-mode images of bladder and corresponding segmented ground truth bladder wall masks along with predicted masks obtained by the proposed network and existing networks.

**TABLE I:** Ablation Study With Respect to the Proposed Method With Respect to Different Loss Weights Selected for Bladder Wall Segmentation (Cross-Validated Metrics (Mean (Std.)))

$\lambda_{1}$	$\lambda_{2}$	$\lambda_{3}$	Dice	MCC	F1	Pr	Se	JI	HD95(mm)
1.0	1.0	0.0	0.78 (0.03)	0.78 (0.02)	0.80 (0.01)	0.79 (0.02)	0.79 (0.03)	0.70 (0.04)	6.2 (2.7)
1.0	1.0	0.25	0.79 (0.02)	0.79 (0.02)	0.81 (0.01)	0.79 (0.03)	0.82 (0.02)	0.73 (0.02)	4.4 (3.1)
**1.0**	**1.0**	**0.5**	**0.82** (0.02)	**0.82** (0.01)	**0.83** (0.02)	**0.82** (0.03)	**0.85** (0.01)	**0.74** (0.03)	**3.5** (2.4)
1.0	1.0	0.75	0.80 (0.02)	0.80 (0.02)	0.81 (0.02)	0.80 (0.03)	0.83 (0.02)	0.72 (0.04)	**3.25** (2.6)
1.5	1.0	0.5	**0.82** (0.02)	0.82 (0.04)	0.82 (0.05)	0.82 (0.01)	0.85 (0.01)	0.73 (0.01)	3.80 (3.1)
0.5	1.0	0.5	0.79 (0.03)	0.79 (0.03)	0.80 (0.02)	0.80 (0.02)	0.83 (0.01)	0.70 (0.02)	3.70 (3.2)
1.0	1.5	0.5	**0.82** (0.02)	0.82 (0.02)	0.82 (0.03)	0.81 (0.03)	0.85 (0.01)	0.74 (0.02)	3.5 (2.5)
1.0	0.5	0.5	0.80 (0.02)	0.80 (0.02)	0.80 (0.02)	0.79 (0.03)	0.84 (0.01)	0.71 (0.04)	3.5 (3.6)

**TABLE II:** Comparative Analysis of State-of-The-Art Methods With Respect to the Proposed Method for Bladder Wall Segmentation (Cross-Validated Metrics (Mean (Std.)))

Method (Ref.)	*Dice*	*MCC*	*F*1	*Pr*	*Se*	*JI*	*HD*95 (mm)	*nFLOPs* (GMac)
U-Net [13]	0.77 (0.05)	0.77 (0.06)	0.78 (0.03)	0.73 (0.03)	0.79 (0.04)	0.64 (0.02)	6.2 (4.5)	397.65
TransU-Net [22]	0.72 (0.03)	0.72 (0.04)	0.72 (0.03)	0.70 (0.02)	0.77 (0.03)	0.62 (0.04)	21 (9.2)	261.36
ResU-Net [34]	0.79 (0.02)	0.79 (0.03)	0.78 (0.03)	0.76 (0.04)	0.80 (0.03)	0.69 (0.05)	4.4 (3.6)	315.95
R2-U-Net [35]	0.49 (0.05)	0.49 (0.05)	0.48 (0.05)	0.43 (0.04)	0.66 (0.04)	0.51 (0.05)	11.2 (7.4)	219.88
Attention-U-Net [36]	0.78 (0.02)	0.78 (0.02)	0.79 (0.03)	0.77 (0.02)	0.83 (0.01)	0.70 (0.03)	4.5 (3.4)	223.31
SwinU-Net [23]	0.79 (0.03)	0.79 (0.02)	0.80 (0.02)	0.75 (0.03)	0.79 (0.02)	0.72 (0.03)	5.2 (3.3)	301.12
Dual Self-Distillation-U-Net [37]	0.78 (0.03)	0.78 (0.01)	0.78 (0.02)	0.73 (0.03)	0.79 (0.02)	0.70 (0.03)	4.1 (2.3)	401.52
nnU-Net [20]	**0.83** (0.03)	**0.83** (0.02)	**0.84** (0.01)	0.79 (0.03)	0.84 (0.02)	**0.74** (0.02)	**3.5** (2.2)	162.12
**Proposed**	**0.82** (0.02)	**0.82** (0.01)	**0.83** (0.02)	**0.82** (0.03)	**0.85** (0.01)	**0.74** (0.03)	**3.5** (2.4)	**13.85**

**TABLE III:** Statistical Significance Analysis of Per-Fold Dice Scores Between the Proposed Method and Baseline Models. Paired T-Tests and Wilcoxon Signed-Rank Tests Were Used (Significance Threshold: p<0.05).

Comparison	Paired t-test p	Wilcoxon p
Proposed vs. U-Net	0.020	0.035
Proposed vs. ResU-Net	0.045	0.055
Proposed vs. R2U-Net	9 × 10^−6^	2 × 10^−5^
Proposed vs. TransU-Net	0.003	0.004
Proposed vs. AttentionU-Net	0.035	0.042
Proposed vs. SwinU-Net	0.040	0.056
Proposed vs. Dual Self Distillation-U-Net	0.040	0.062
**Proposed vs. nnU-Net**	**0.310**	**0.340**

## Appendix

**TABLE IV:** Summary of Notations Used in This Work

Notation	Denoted as
$x_{1},y_{1}$	Horizontal, vertical axis coordinate of the upper bladder wall
$x_{2},y_{2}$	Horizontal, vertical axis coordinate of the lower bladder wall
a,b	Quadratic, linear coefficient of the polynomial to fit the bladder wall contour
c	Intercept term of the polynomial to fit the bladder wall contour
θ	Parameter which is half the thickness of the bladder wall.
$n_{t},n_{b}$	Total number of labeled points in upper and lower bladder wall respectively
N	Number of filters/kernels
I	Input image
V	Number of channels in the input image
A,B	Spatial dimensions of the input image
K	Kernel/Filter
F	Filter dimension
j	Index variable belonging to number of filters
v	Index variable belonging to number of channels
w	Weight vector
*	Convolution operation
O	Convolved output
O′	Convolved output after re-arrangement
$A_{5}$	Output feature map obtained using a 5 × 5 convolution
$A_{D3}$	Output feature map obtained using a 3-rate dilated 3 × 3 convolution
$O_{G}$	Output feature map obtained after global average pooling operation
$O_{D1}$	Output feature map obtained after first dense layer
$\sigma_{G}$	Gaussian error linear unit activation
$O_{D1^{\prime }}$	Output feature map obtained after passing $O_{D1}$ through $\sigma_{G}$
$W_{\text{De}}$	Weight vector of dense layer
$O_{D2}$	Output feature map obtained after second dense layer
$O_{\text{AA}}$	Output feature map obtained after adaptive attention unit
$\sigma_{s}$	Sigmoid activation
α	Scale factor obtained after sigmoid activated output
⊗	Element-wise multiplication
P	Predicted probability map
G	Binary ground truth mask
${ℒ}_{\text{Dice}},{ℒ}_{\text{Focal}},{ℒ}_{\text{Boundary}}$	Dice, Focal, and boundary losses
$\lambda_{1},\lambda_{2},\lambda_{3}$	Attention weights for dice, focal, and boundary/edge loss terms respectively
